# Identification of Fungal Pathogens of Chinese Chestnut Fruit Rot and Analysis of Resistance Differences Among Major Cultivars

**DOI:** 10.3390/microorganisms14010113

**Published:** 2026-01-05

**Authors:** Haijiao Xu, Wenshi Zhao, Yan Guo, Jianchao Cui, Gang Niu, Shuhang Zhang, Ying Li, Litao Li, Rui Jiao, Xumin Wang, Guangpeng Wang, Limin He

**Affiliations:** Changli Institute of Pomology, Hebei Academy of Agriculture and Forestry Sciences, Changli 066600, China; xuhaijiao1234@sina.cn (H.X.); zhaows1102@163.com (W.Z.); guoyan@126.com (Y.G.); cjc19880320@126.com (J.C.); niug@nwafu.edu.cn (G.N.); zhangshuhang1203@163.com (S.Z.); beierdina3320@sina.com (Y.L.); wangyillt126@126.com (L.L.); jiaozhirui@163.com (R.J.); 18931353275@163.com (X.W.)

**Keywords:** chestnut fruit rot, *Castanea mollissima*, morphological identification, phylogenetics, multi-pathogen infections, resistance differences, correlation analysis, soluble sugar

## Abstract

This study aimed to identify fungal species causing fruit rot of chestnut (*Castanea mollissima* Blume) in Hebei Province, China and analyze the resistance differences among major cultivars. A total of 220 fungal isolates were obtained from healthy and diseased kernels, which were classified into six distinct genera: *Diaporthe* (48.6%), *Talaromyces* (22.3%), *Alternaria* (10.5%), *Mucor* (9.5%), *Fusarium* (5.5%), and *Rhizopus* (3.6%). Based on both morphological and molecular analyses, six representative isolates of the six genera were identified as *Diaporthe eres* Nitschke, *Talaromyces rugulosus* Samson, N. Yilmaz, Frisvad & Seifert, *Alternaria alternata* (Fr.) Keissl., *Mucor circinelloides* Tiegh., *Fusarium proliferatum* (Matsush.) Nirenberg, and *Rhizopus stolonifer* (Ehrenb.) Vuill. Among these, *D. eres* was first reported to cause fruit rot on *C. mollissima* in China. Moreover, disease resistance evaluation of major cultivars showed significant differences: YG, YSSF, and DBH exhibited strong resistance under both natural conditions (with 1.67% to 5.27% DI after 180 days storage) and artificial inoculation (with 32.96 ± 0.64 to 52.61 ± 0.55 DI); while YJ was highly susceptible (with 47.71% decay incidence and 70.50 ± 7.22 DI). Correlation analysis revealed that the disease index was negatively correlated with sucrose and sorbitol contents, but positively correlated with stachyose and fructose contents. This study advances the understanding of postharvest chestnut fruit rot and provides a theoretical basis for breeding resistant cultivars and developing control strategies to mitigate losses and ensure food safety.

## 1. Introduction

The Chinese chestnut (*Castanea mollissima* Blume), a key economic forest species, is widely cultivated throughout China and was introduced to Europe in the 19th century [1]. The nuts are rich in starch, protein, functional polysaccharides, essential fatty acids, vitamins, and minerals, which impart various health benefits, such as lowering cholesterol, preventing obesity and diabetes, combating tumors, and enhancing immunity [2]. This importance is reflected in China’s status as the world’s largest producer, with an annual yield exceeding 1.52 million tons since 2023 [3]. However, chestnuts are prone to rot and deteriorate due to microbial infection during the harvest and postharvest storage periods, leading to significant quality and production losses [4]. In China, improper storage of chestnuts results in annual losses amounting to 20–30% of the total production, and even exceeds 50% in several production areas by the late storage [5]. Moreover, pathogens also produce mycotoxins (e.g., aflatoxins, ochratoxins, fumonisins, and T-2/HT-2 toxins), posing a serious food safety threat [4]. Hence, it is crucial to clarify the incidence of rot disease and the species of pathogens in different chestnut cultivars for developing targeted control strategies.

Various pathogens have been reported to cause chestnut fruit rot during preharvest and postharvest storage [2]. Apart from two bacterial species, *Brevundimonas vesicularis* (Busing et al.) Segers et al. [6] and *Pseudomonas amygdali* pv. *aesculi* Mull. Arg. [7], the disease primarily results from complex infections of multiple pathogenic fungi, many of which exhibit latent colonization. Reported fungal genera include *Alternaria*, *Botryosphaeria*, *Colletotrichum*, *Diaporthe*, *Fusarium*, *Penicillium*, *Rhizopus*, *Trichothecium*, *Sclerotinia*, *Gnomoniopsis*, *Phomopsis*, *Acrospeira*, *Aspergillus*, *Botrytis*, *Diplodia* and *Cladosporium* [4,8,9]. A total of 308 fungal operational taxonomic units were detected during chestnut postharvest storage in Italy chestnut fruits, with new contaminants emerging in specific phases such as the ‘cold bath’ and ‘storage’ [4]. Nevertheless, the identity and pathogenicity of the predominant fungi among them have not been identified.

Furthermore, pathogen types and community structure are strongly influenced by geographic origin and cultivar differences [10]. For instance, *Gnomoniopsis smithogilvyi* L.A. Shuttlew., E.C.Y. Liew & D.I. Guest was first reported as a nut rot pathogen in Australian *Castanea sativa* Mill. [11]; whereas, *Gnomoniopsis clavulata* (Ellis) Sogonov and *Gnomoniopsis paraclavulata* Sogonov were documented only in the USA, and *Gnomoniopsis daii* C.M. Tian & N. Jiang was described as a novel pathogen affecting *C. mollissima* in China [8]. Additionally, cultivar characteristics (e.g., skin structure, fruit firmness, and sugar content) also significantly influence pathogen infection and spread [12]. However, current studies on the diversity of chestnut fruit pathogens remain narrowly focused on single cultivars and specific production regions [11,12], neglecting the resistance differences among various chestnut cultivars and the mechanisms of pathogens’ complex infections, thereby complicating the development of effective control strategies.

Chestnuts from the Yanshan area of Hebei Province, as the largest contiguous cultivation belt in China, hold a prestigious position in the global market [13]. During the surveys of chestnut rot conducted in Hebei province in China, typical fruit rot symptoms were observed. Nonetheless, the major pathogenic fungal species in this area remain unknown. Our aim in this study was to identify the pathogenic species of chestnut fruit rot and to analyze the disease resistance differences among major cultivars in this region. These findings will offer a theoretical basis to inform the breeding of rot-resistant chestnut cultivars and the creation of sustainable postharvest strategies, which are crucial for minimizing losses and ensuring food safety.

## 2. Materials and Methods

### 2.1. Disease Surveys and Isolation of Fungi

Chestnuts cv. Dabanhong (DBH), Yanguang (YG), Yanshan Shuofeng (YSSF), Yanshan Zaofeng (YSZF), Yanjing (YJ), and Yanli1 (X19-94) are the main cultivars in the Yanshan area. All these cultivars were grafted onto three-year-old YSZF seedling rootstocks in 2004, with five trees per cultivar planted at a 3 m × 3 m spacing and managed under standard horticultural practices. The fruits were separately harvested at the mature stage based on uniform size and free of defects in the Chestnut Germplasm Repository of the Changli Institute of Pomology, Hebei Academy of Agriculture and Forestry Sciences (119°15′ E, 39°72′ N) [14]. The collected fruits were packaged in PE bags and stored at 4 °C for further experimentation.

Chestnut rot development was assessed at 30-day intervals over a 180-day cold storage (4 °C) period. The chestnut decay incidence (Equation (1)) was recorded as the percentage of decayed nuts among 200 randomly selected fruits per cultivar, with three replicates. Chestnuts exhibiting typical symptoms were collected and photographed. Additionally, five healthy fruits were randomly sampled at each time point for tissue isolation.

(1) $$Decay\;incidence=\frac{Numbers\;of\;decay\;chestnuts}{Total\;numbers\;of\;chestnuts}\times 100\%$$

Fungi were isolated following Chen et al. [15] with minor modifications. Tissue segments (5 mm × 5 mm) were excised from both healthy and decayed chestnuts at each sampling time. The tissues were surface-sterilized in 70% ethanol for 1 min, followed by 1% sodium hypochlorite for 5 min, rinsed thoroughly with sterile distilled water, and placed on potato dextrose agar (PDA). After incubating at 25 °C in the dark, isolates were purified by transferring single hyphal tips to new PDA plates. The resulting cultures were incubated under the same conditions for approximately 7 days and subsequently stored at 4 °C [16].

### 2.2. Morphological Identification and Characterization

Morphological characterization of the isolates was performed on sporulating cultures grown on PDA at 25 °C under a 12 h light regime for 7 days. Cultural characteristics, including colony color and texture, were documented. Colony diameters were measured daily to determine growth rates. Micromorphological features (such as hyphae, conidia, etc.) were observed using an Olympus BX53 optical microscope equipped with an Olympus DP74 color camera (Olympus, Tokyo, Japan); 200 conidia per isolate were randomly measured.

### 2.3. DNA Extraction and Phylogenetic Analysis

Genomic DNA was extracted from 7-day-old mycelium grown on PDA using a Super Plant Genomic DNA DP360 Kit (Tiangen Biotech, Beijing, China) according to the manufacturer’s instructions. The rDNA-ITS (ITS) region was amplified with primers ITS1/ITS4 (Table S1) for preliminary identification [17]. For precise species identification, multiple gene regions (Table S1) were amplified and sequenced [17,18,19,20]. The PCR mixture and conditions for all gene regions followed established methods [8,15]. Amplification products were verified by 1% agarose gel electrophoresis and sequenced by a commercial provider (Beijing Tsingke Biotech Co., Ltd., Beijing, China).

Sequence queries were conducted using the NCBI BLASTn 2.17.0. Phylogenetic analyses incorporated additional sequences from GenBank (Tables S2–S6). Sequences for each locus were aligned with ClustalX [21], manually refined in MEGA7.0 [22], and then concatenated in BioEdit [23]. Phylogenetic trees were reconstructed using the Neighbor-joining (NJ) method with 1000 bootstrap replicates in MEGA7.0.

### 2.4. Pathogenicity Testing

Conidia were prepared according to Jia et al. [24] with minor modifications. After incubation on PDA at 25 °C for 14 days, conidia were harvested by scraping into a sterile 0.02% (*v*/*v*) Tween 80 solution. The suspension was filtered through three layers of mirror cleaning paper to remove mycelial and agar. The conidial concentration was determined and adjusted to 2 × 10^6^ conidia/mL using a hemocytometer. Fresh conidial suspensions were prepared individually for each fungal species.

For pathogenicity assays [2], healthy and uniformly sized chestnuts (cv. YSZF) were surface-sterilized by immersion in 1% sodium hypochlorite for 10 min, rinsed thoroughly with sterile distilled water, and air-dried on sterile filter paper. A single wound (0.5 mm diameter, 5 mm depth) was made on each chestnut. Each wound was inoculated with 20 μL of the conidial suspension of a single isolate, with an equal volume of sterile 0.02% (*v*/*v*) Tween 80 solution serving as the control. All fruits were placed in a moist chamber and kept at 25 °C for 15 days under a 12 h light/12 h dark photoperiod. The experiment was conducted twice with three replicates (10 chestnuts each) per fungi species. To fulfill Koch’s postulates, we re-isolated the pathogens from the inoculated chestnuts and confirmed that they were identical to the original strains through morphological and molecular analysis as described above.

### 2.5. Assessment of Disease Severity on Chestnut Cultivars

A combined inoculum was prepared by mixing equal volumes of fresh conidial suspensions (2 × 10^6^ conidia/mL) from representative strains of each pathogenic species. The six chestnut cultivars, DBH, YG, YSSF, YSZF, YJ, and X19-94 stored at 4 °C for 40 days after harvest, were surface-sterilized and inoculated with 20 μL of the combined inoculum, following the incubation conditions as described above.

According to Çakar et al. [25], with minor modifications, disease severity was quantified 15 days after inoculation by measuring lesion diameters. Each chestnut was assessed for severity on a 0–9 scale, in which 0, no lesion; 1, lesion ≤ 1.5 mm; 3, 1.5 mm < lesion ≤ 5 mm; 5, 5 mm < lesion ≤ 10 mm; 7, 10 mm < lesion ≤ 20 mm; and 9, lesion > 20 mm or complete rot. The disease index (DI) was calculated using the following equation (Equation (2)):

(2) $$DI=\frac{X_{0}\times 0+X_{1}\times 1+X_{3}\times 3+X_{5}\times 5+X_{7}\times 7+X_{9}\times 9}{X_{0}+X_{1}+X_{3}+X_{5}+X_{7}+X_{9}}\times 100$$

where X_0_, X_1_, X_3_, X_5_, X_7_, and X_9_ represent the number of chestnuts with severity scores of 0, 1, 3, 5, 7, and 9, respectively. Each treatment investigated 50 fruits, and the experiment was conducted twice with three replicates per treatment.

### 2.6. Statistical Analysis

All data were expressed as means ± standard deviation and analyzed with three replicates per treatment at each time point. For each time, statistical analysis was performed using one-way analysis of variance (ANOVA) following Duncan’s multiple range test at a 95% confidence interval for mean comparisons. The correlation was evaluated with the Pearson correlation coefficient (r) following the quality data of chestnuts in our previous studies [26]. All analyses were conducted using SPSS software (version 22.0, IBM Corp., Armonk, NY, USA).

## 3. Results

### 3.1. Disease Symptom and Development During Postharvest Storage

**Figure 1:**
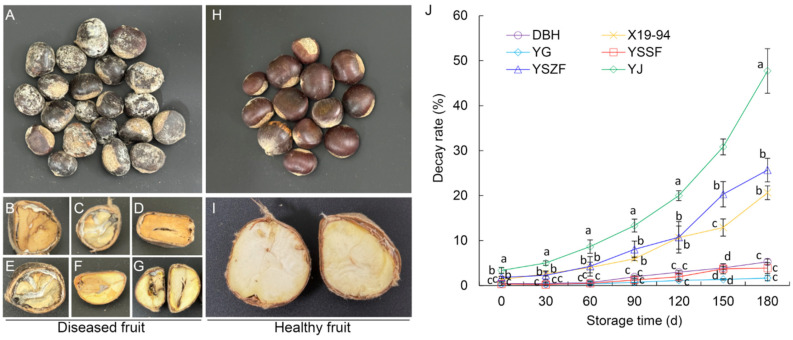
Disease symptoms and decay incidence of Chinese chestnut rot. (**A**–**I**) Naturally rotted chestnuts (**A**–**G**) and healthy chestnuts (**H**,**I**) were compared with representative symptoms on the external surface (**A**,**H**) and internal cross-section (**B**–**G**,**I**). (**J**) Decay incidence in six chestnut cultivars during cold storage. Data are expressed as the mean ± SD. Different letters in each storage period indicate significant differences at *p* < 0.05.

During cold storage, diseased chestnuts exhibiting a range of typical symptoms were observed across all cultivars. Superficially, the outer and inner seed coats generally remained intact, though the inner coats occasionally darkened or brownish-black. Internally, dark brown to black necrotic lesions developed on the kernel surface, often penetrating deeply into the tissue. Cross-sections frequently revealed grayish-white to dark gray, streaked cavities. In severe cases, the shells lost their luster and were covered with conspicuous grayish-white or olive-green mold. The kernels became shriveled and exhibited either dry or soft rot, with internal tissues turning yellowish-brown or grayish-black (Figure 1A–G). In contrast, healthy chestnuts displayed plump, glossy shells and firm kernels of uniform milky-white to pale-yellow color (Figure 1H,I).

Significant differences in decay development were observed among the chestnut cultivars over a 180-day storage period. At the initiation of storage (0 d), cultivar YJ showed the highest decay incidence (3.36%), followed by YSZF (1.83%) and X19-94 (1.33%), while DBH, YSSF, and YG were all below 0.45%. As storage progressed, decay incidence increased in all cultivars, though the rate of increase and final severity varied markedly. By day 180, YJ reached the highest incidence (47.71%), whereas YSZF and X19-94 showed moderate levels of 25.67% and 20.64%, respectively. In contrast, DBH, YSSF, and YG consistently maintained the lowest decay incidence throughout storage, with final incidence ranging only from 1.67% to 5.27% at 180 days (Figure 1J, Table S7). These results indicate that YG, YSSF, and DBH are relatively resistant to chestnut decay, whereas YSZF and X19-94 exhibit intermediate susceptibility, and YJ is highly susceptible.

### 3.2. Fungal Isolation and Identification

Although not all surface-sterilized tissue segments yielded fungal growth, a total of 220 fungal isolates (23 from healthy kernels and 197 from diseased kernels) were obtained from 35 healthy and 55 diseased samples collected during cold storage. Preliminary identification was based on colony morphology, conidial characteristics on PDA, and ITS sequence analysis. As a result, all isolates were classified into six genera: *Diaporthe* (48.6% in total isolates, 11 from healthy/96 from diseased samples), *Talaromyces* (22.3%, 3/46), *Alternaria* (10.5%, 1/22), *Mucor* (9.5%, 2/19), *Fusarium* (5.5%, 4/8), and *Rhizopus* (3.6%, 2/6). All genera were isolated from both healthy and diseased tissues. Based on their similar cultural and morphological characteristics, and ITS assay results, six representative isolates (BL-1 to BL-6), representing each of the six genera, were selected for further study.

### 3.3. Molecular Identification and Phylogenetic Analysis

**Figure 2:**
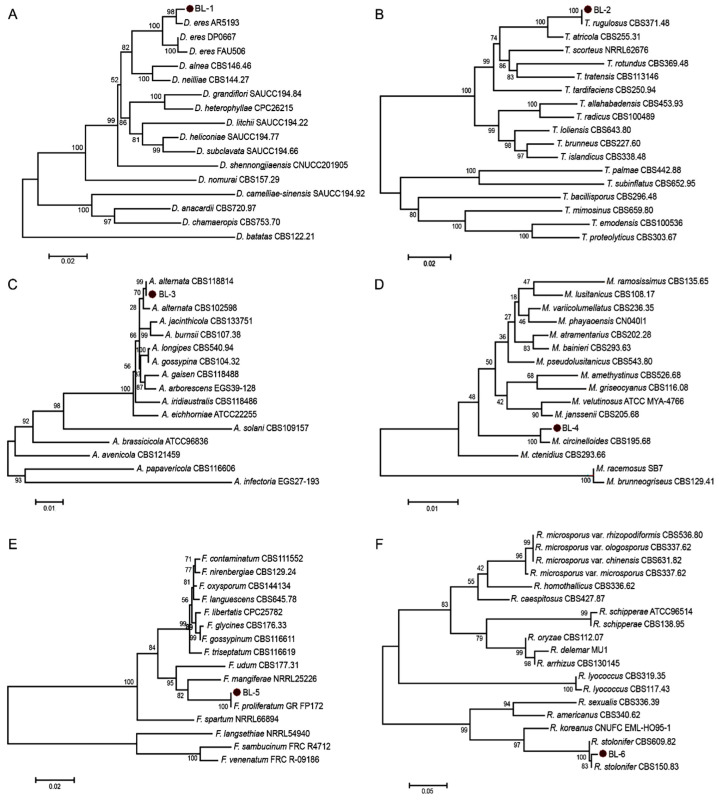
Phylogenetic analysis of chestnut fruit rot pathogens based on the NJ method. (**A**) Multi-locus phylogeny of BL-1 and related *Diaporthe* species inferred from combined ITS, TUB, TEF, CAL, and HIS sequences. Aligned gene boundaries spanned 1–551 bp, 552–998 bp, 999–1377 bp, 1378–1806 bp, and 1807–2312 bp, respectively. Scale bar indicates 0.02 substitutions per nucleotide position. (**B**) Multi-locus phylogeny of BL-2 and *Talaromyces* species (ITS: 1-601, TUB: 602-1049, RPB2: 1050-1852). Scale bar: 0.02. (**C**) Multi-locus phylogeny of BL-3 and *Alternaria* species (ITS: 1-555, GAPDH: 556-1138, TEF:1139-1386, RPB2: 1387-2110). Scale bar: 0.01. (**D**) ITS-based phylogeny of BL-4 and related *Mucor* species. Scale bar: 0.01. (**E**) Multi-locus phylogeny of BL-5 and *Fusarium* species (RPB2: 1-787, TEF: 788-1426). Scale bar: 0.02. (**F**) ITS-based phylogeny of BL-6 and *Rhizopus* species. Scale bar: 0.05. Bootstrap support values (1000 repetitions) are indicated at nodes. The six isolates analyzed in this study (BL-1 to BL-6) are emphasized with red dots.

The six representative isolates were sequenced and subjected to phylogenetic analysis using the Neighbor-joining (NJ) method with either combined multi-locus sequences or the ITS region alone. As shown in Figure 2A, a multi-locus phylogenetic analysis of the BL-1 isolate and related *Diaporthe* species was processed based on ITS, TUB, TEF, CAL, and HIS gene regions, with a total alignment length of 2312 characters including gaps. BL-1 clustered with reference strains of *Diaporthe eres* Nitschke (including AR5193) with 93% bootstrap-support, confirming its identification as *D. eres*. For BL-2, multi-locus analysis of ITS, TUB, and RPB2 (1852 characters) showed clustering with *Talaromyces rugulosus* Samson, N. Yilmaz, Frisvad & Seifert (CBS371.48) with 100% bootstrap-support, identifying it as *T. rugulosus* (Figure 2B). Similarly, BL-3 (2110 characters from ITS, GAPDH, TEF, and RPB2) clustered with *Alternaria alternata* (Fr.) Keissl. (CBS118814) under 99% bootstrap-support value, confirming its identification as *A. alternata* (Figure 2C). In the case of BL-5, BLAST analysis of the ITS region showed 99.79% sequence similarity to *Fusarium proliferatum* (Matsush.) Nirenberg (isolate MT-S-1, IGPEB-SH11, DHHJYK2 et al.), *Fusarium oxysporum* Schltdl. (A221), and *Fusarium fujikuroi* Nirenberg (FPM27); phylogenetic analysis based on combined RPB2 and TEF sequences (1426 characters) further placed the isolate within the *F. proliferatum* clade with 100% bootstrap support relative to the reference stain GR_FP172, establishing its identity as *F. proliferatum* (Figure 2E). For BL-4 and BL-6, ITS sequences showed up to 97% identity with reference sequences of *Mucor circinelloides* Tiegh. and *Rhizopus stolonifer* (Ehrenb.) Vuill. in the NCBI database, respectively. Phylogenetic analysis of ITS region confirmed that BL-4 formed a well-supported clade with *M. circinelloides* CBS526.68 (Figure 2D), while BL-6 clustered with *R. stolonifer* CBS150.83 and CBS609.82 under 83% and 100% NJ bootstrap-support, respectively (Figure 2F). These results support the identification of BL-4 as *M. circinelloides* and BL-6 as *R. stolonifer*.

### 3.4. Morphology and Taxonomy

***Diaporthe eres*** Nitschke (1870) [15].

Index Fungorum No.: 802934.

**Figure 3:**
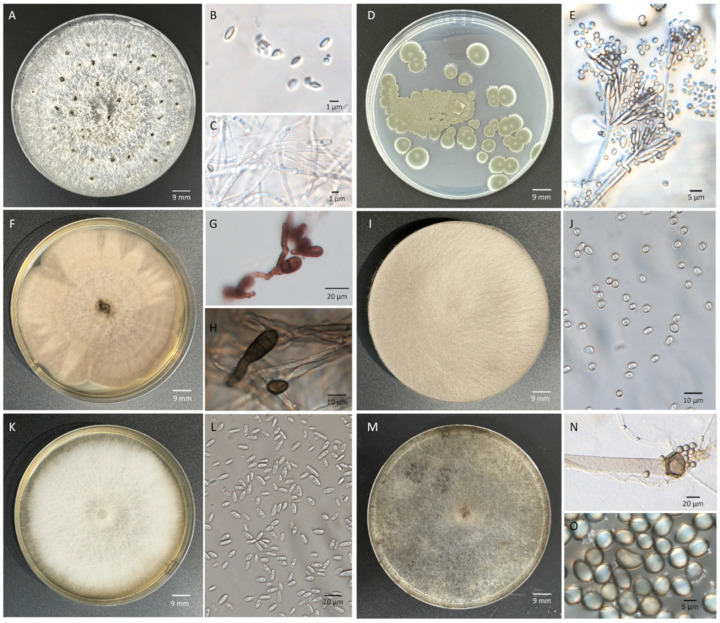
Morphological characters of pathogens isolated from diseased chestnuts. (**A**–**C**) *D. eres* BL-1: colony (**A**), conidia (**B**) and hyphae (**C**). (**D**,**E**) *T. rugulosus* BL-2: colony (**D**), conidiophores and conidia (**E**). (**F**–**H**) *A. alternata* BL-3: colony (**F**), conidiophores (**G**), conidia and hyphae (**G**,**H**). (**I**,**J**) *M. circinelloides* BL-4: colony (**I**) and conidia (**J**). (**K**,**L**) *F. proliferatum* BL-5: colony (**K**) and conidia (**L**). (**M**–**O**) *R. stolonifer* BL-6: colony (**M**), hyphae, sporangiophores and sporangia (**N**), and sporangiospores (**O**). All colonies were cultured on PDA for 7 days at 25 °C under a 12 h photoperiod.

Description of isolate BL-1: Colonies on PDA at 25 °C under a 12 h light regime exhibited a growth rate of 14.8 ± 0.6 mm/d. Aerial mycelium initially was white and fluffy, later developed white-cream to light brown, cottony, mycelial mats, and eventually produced gray to black pycnidia, and no soluble pigment. Hyphae were hyaline, smooth-walled, septate, and branched. Alpha conidia were hyaline, aseptate, ellipsoidal to fusiform, measuring 3.3–8.5 μm × 0.9–3.1 μm (mean ± SD = 5.2 ± 1.7 μm × 2.1 ± 0.8 μm, *n* = 200). Beta conidia were not observed (Figure 3A–C).

***Talaromyces rugulosus*** (Thom) Samson, N. Yilmaz, Frisvad & Seifert (2011) [27]

Index Fungorum No.: 560672.

Description of isolate BL-2: Colonies on PDA were plane with a white margin, regular edge, green to dull green conidia pile, exudates and soluble pigment absent at 25 °C. Hyphae appeared hyaline, smooth, septate, and branched. Conidiophores were biverticillate with smooth-walled stipes and unswollen tops, 3–6 metulae (6.5–13.5 μm × 2.3–4.0 μm), 3–8 acerose phialides (7.7–14.0 μm × 1.3–2.5 μm) per metulae. Conidia were gray-green in color, smooth-walled, ellipsoidal or round, 1.5–5.5 µm × 1.5–3.0 µm (3.0 ± 0.9 µm × 2.4 ± 0.4 µm) (*n* = 200). Ascomata were not observed (Figure 3D,E).

***Alternaria alternata*** (Fr.) Keissl. (1912) [28]

Index Fungorum No.: 119834.

Description of isolate BL-3: Colonies on PDA at 25 °C exhibited a growth rate of 10.2 ± 1.5 mm/d. They were initially grayish-white, later developing a pale gray to gray-brown base, occasionally with brownish center zones, without exudates. Abundant aerial mycelium extended from the center, forming distinct radial surface patterns. Hyphae were pale brown to brown, smooth, septate, and branched. Conidiophores were pale to brown, smooth, septate, solitary or clustered, straight or flexuous, and 32.6–85.3 µm × 1.8–4.2 µm (59.9.2 ± 13.8 µm × 3.1 ± 0.7 µm, *n* = 50). Conidia were ellipsoid to ovoid or obclavate to obpyriform, brown, smooth, measuring 8.3–27.5 µm × 4.2 to 14.5 µm (18.7 ± 5.6 µm × 11.4 ± 2.6 µm, *n* = 200), with 0–2 longitudinal and 1–5 transverse septa, beakless or bearing a light-brown, cylindrical to tapered beak (Figure 3F–H).

***Mucor circinelloides*** Tiegh. (1875) [29]

Index Fungorum No.: 198947

Description of isolate BL-4: Colonies grew rapidly on PDA at 25 °C, reaching a growth rate of up to 21.1 ± 1.6 mm/d. Abundant aerial mycelium exhibited velvety texture and light gray, most frequently reaching the lid of the Petri dish, exudates and soluble pigment absent. Hyphae appeared hyaline, smooth, without non-septate, branched. Sporangiospores were globose or ellipsoidal, hyaline, smooth, small, 2.6–5.8 µm × 2.2–5.5 µm (4.0 ± 0.8 µm × 3.3 ± 0.7 µm, *n* = 200) (Figure 3I,J).

***Fusarium proliferatum*** (Matsush.) Nirenberg (1976) [30]

Index Fungorum No.: 362256

Description of isolate BL-5: On PDA at 25 °C, colonies grew at a rate of 11.1 ± 0.9 mm/d, producing abundant, white, villous aerial mycelium, with regular margins, exudates absent and no zonation or violet pigmentation. Hyphae appeared hyaline, smooth, septate, and branched. Microconidia were abundant, hyaline, smooth, 0-septate, oval, elliptical or club, 7.4–15.4 µm × 2.3–7.4 µm (11.5 ± 2.0 µm × 4.8 ± 1.0 µm, *n* = 200). Macroconidia were not observed on PDA (Figure 3K,L).

***Rhizopus stolonifer*** (Ehrenb.) Vuill. (1902) [31]

Index Fungorum No.: 417250

Description of isolate BL-6: On PDA at 25 °C, colonies grew rapidly at a growth rate of 37.7 ± 2.5 mm/d, reaching the lid of the Petri dish within 24 h. Abundant aerial mycelium was dense and fluffy, changing from initially white and then gray-white to black-brown, with no exudates observed. Hyphae were broad, ribbon-like, branched, coenocytic, and aseptate. Sporangiophores were unbranched, yellowish-brown to dark brown, erect, and terminating in globose sporangia. Sporangia were spherical, initially white, and then black, and contained a large number of sporangiospores and a columella. Sporangiospores were non-motile, hyaline to pale brown, in various forms (spherical, ellipsoidal and angular), non-septate, with continuous and obvious ridges on the surface along the spores, 6.4–9.6 µm × 5.8–8.4 μm (8.3 ± 1.0 µm × 6.7 ± 0.7 µm, *n* = 200) (Figure 3M,O).

### 3.5. Pathogenicity Study

**Figure 4:**
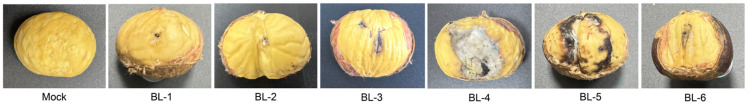
Pathogenicity of the six isolates to *C. mollissima* cv. YSSF. Chestnut fruits were wounded inoculated without (Mock) and with 2 × 10^6^ conidia/mL of the fungal isolates (BL-1 to BL-6), respectively. Inoculated nuts were incubated in a moist chamber and kept at 25 °C for 15 days with a 12-h photoperiod. Representative nuts were photographed at 15 d after inoculation. The experiment was repeated twice and similar results were observed.

After 15 days of inoculation on wounded nuts (cv. YSSF), all six isolates, namely *D. eres* (BL-1), *T. rugulosus* (BL-2), *A. alternata* (BL-3), *M. circinelloides* (BL-4), *F. proliferatum* (BL-5), and *R. stolonifer* (BL-6), caused brown to black rot symptoms, with the artificially infected incidence of 100%. Among them, BL-4 and BL-5 exhibited the strongest virulence, characterized by extensive grayish-white mycelium with rot or large black-brown rotting lesions with deep necrosis; BL-3 and BL-6 displayed moderate virulence, presenting localized brown necrotic spots; while BL-1 and BL-2 demonstrated relatively weakest virulence, forming small lesions at the inoculation site. None of the control nuts showed any disease or symptoms (Figure 4). The pathogens were re-isolated from the inoculated chestnuts after showing typical symptoms, satisfying Koch’s postulates.

### 3.6. Disease Severity of Various Chestnut Cultivars

**Figure 5:**
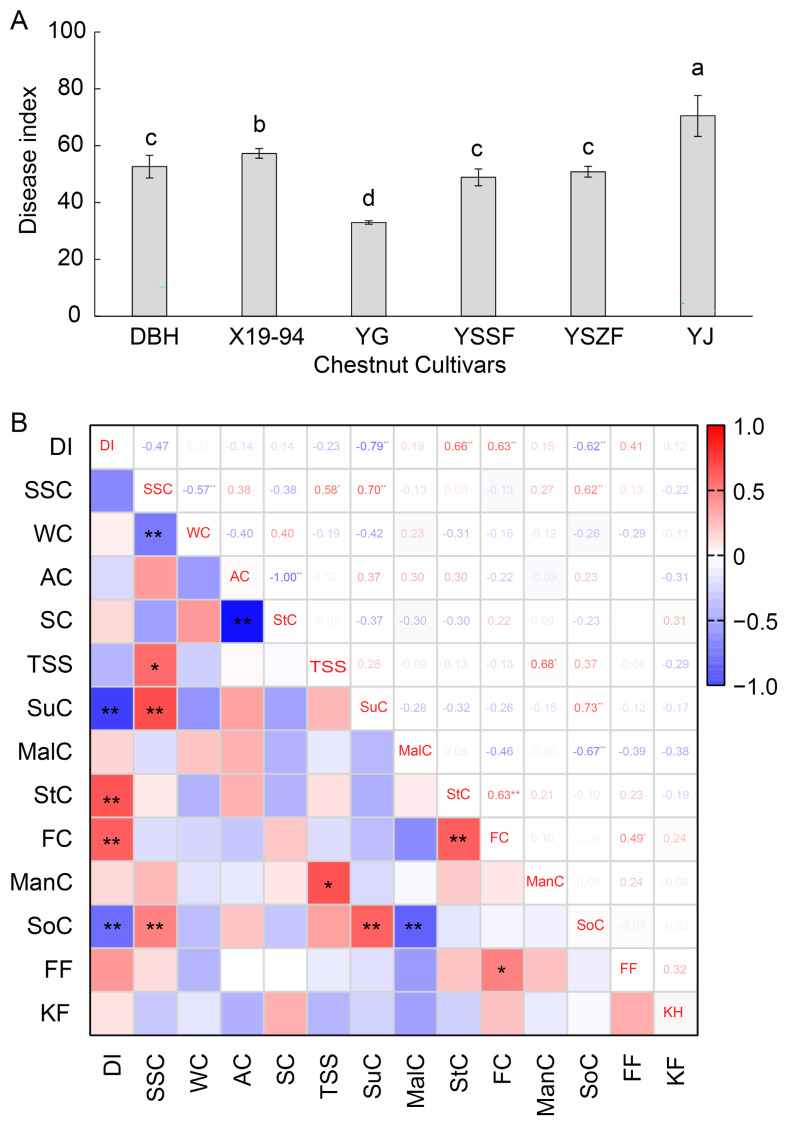
Disease index of chestnut inoculated with mixed fungi (**A**) and correlation analysis between the disease index and relevant contents in chestnut samples stored for 40 days (**B**). (**A**) Following 40 days of postharvest storage at 4 °C, nuts of the six chestnut cultivars (DBH, YG, YSSF, YSZF, YJ, and X19-94) were inoculated with the combined inoculum. The disease index was determined at 15 d after inoculation by measuring the lesion diameter of 50 nuts in each cultivar with three replicates. Different letters indicate significant differences at *p* < 0.05. (**B**) The content of soluble sugars and firmness data of the chestnut cultivars, following 40 days of postharvest storage at 4 °C, are listed in Table S8. DI: disease index, SSC: soluble solid content, WC: water content, AC: amylose content, SC: starch content, TSS: total soluble sugar content, SuC: sucrose content, MalC: maltose content, StC: Stachyose content, FC: fructose content, ManC: mannitol content, SoC: sorbitol content, FF: fruit firmness, KF, Kernel firmness. * and ** indicate significant differences at *p* < 0.05 and *p* < 0.01, respectively.

To evaluate the resistance of various chestnut cultivars to fungal infection, disease indices were assessed following inoculation with the mixed fungi. As shown in Figure 5A, significant differences in disease severity were observed among the chestnut cultivars at 15 days post-inoculation (Figure 5A). The YG cultivar exhibited the strongest resistance, with the lowest disease index of 32.96 ± 0.64. For cultivars YSSF, YSZF and DBH, the disease index had slightly increased (48.87 ± 2.97, 50.83 ± 1.89, and 52.61 ± 0.55, respectively), followed by X19-94 (57.29 ± 1.71). In contrast, the disease index of cultivar YJ was highest (70.50 ± 7.22), indicating it was a relatively susceptible cultivar.

To better analyze relative factors of quality that influence chestnut rot disease, correlation analysis was performed between the disease index of chestnuts infected with mixed fungi and soluble sugars, and firmness (Table S8) [26]. As shown in Figure 5B, the disease index of chestnuts showed positive correlations with stachyose content (StC) and fructose content (FC) (*p* < 0.01 and r > 0.5). However, it had negative correlations (*p* < 0.01 and r < −0.5) with sucrose content (SuC) and sorbitol content (SoC), and showed no significant correlation with respiratory intensity.

## 4. Discussion

The accurate identification of fungal pathogens has shifted from morphology-based approaches toward an integrated taxonomic approach centered on multi-locus phylogenetic analysis [32]. This transition addresses the limitations of relying solely on the ITS region as a universal barcode [33]. In this study, ITS variability is insufficient to distinguish closely related species with distinct ecological or pathogenic traits in genera such as *Diaporthe*, *Talaromyces*, *Alternaria*, and *Fusarium*. By combining gene loci with different evolutionary rates—e.g., ITS, GAPDH, TEF1, and RPB2—the multi-locus phylogenetic analysis significantly enhances both the resolution and statistical support of the phylogenetic tree, thereby enabling more precise species delineation [34]. It should be emphasized that molecular data do not replace morphological examination; rather, detailed morphological characterization remains essential for validating phylogenetically defined species boundaries [35]. By adopting this combined approach, we successfully identified these pathogens and overcame the constraints of relying solely on the ITS region, thereby ensuring accurate species identification.

This study systematically revealed the diversity of pathogenic fungi causing postharvest fruit rot in chestnuts during cold storage and identified the resistance differences among various cultivars. While classical chestnut pathogens like *Botryosphaeria*, *Penicillium*, *Colletotrichum*, etc., are well-documented [4], our results indicate that chestnut fruit rot is primarily caused by six pathogenic fungi: *D. eres*, *T. rugulosus*, *A. alternata*, *M. circinelloides*, *F. proliferatum*, and *R. stolonifer*. Among these, the genus *Diaporthe* had the highest isolation frequency (48.6%); one representative isolate was confirmed to be *D. eres*, suggesting it may play a dominant role in postharvest chestnut rot. These differences may be explained by possible geographical, climatic, or host cultivars. Interestingly, previous studies showed that *D. eres* can infect more than 280 hosts, such as walnuts (*Juglans regia* L.) [36], cherry (*Prunus avium* (L.) L.) [15], pear (*Pyrus bretschneideri* Rehder) [37], and persimmon (*Diospyros kaki* Thunb.) [38], and so on. To our knowledge, this is the first report of *D. eres* causing fruit rot on chestnut (*C. mollissima*) in China. Other identified species, such as *A. alternata* and *F. proliferatum*, have also been reported to cause mixed infections in chestnuts, leading to disruptions in the activity of cell wall-degrading enzymes and antioxidant enzymes, as well as in nutrient contents, and physiological levels, and culminating in cell structure disintegration [2]. Genera *Talaromyces*, *Mucor* and *Rhizopus* have been frequently detected as influencing postharvest fruit quality and storability, while their pathogenicity on chestnuts remains undefined [9]. In this study, our pathogenicity assays employed wound-inoculation to confirm the virulence of all six isolated fungi. This approach effectively replicates common postharvest infection routes, such as those initiated by mechanical injury or insect damage [11,39,40]. It should be noted that under natural conditions, these pathogens may also infect via direct penetration through natural openings (e.g., lenticels, micropyles) or even intact fruit surfaces and the flowers, given suitable environmental cues [11]. Notably, all six fungi were isolated from both symptomatic and apparently healthy kernels, indicating that the pathogens may have established latent infection prior to harvest or colonized through wounds or natural openings after harvest [39]. Future studies comparing wound and non-wound inoculation methods would help clarify the primary infection pathways for each species and distinguish between latent colonization and wound-dependent infection. These latent infections likely developed into symptomatic disease during storage, as the physiological state of the fruit changed (e.g., physiological changes and nutritional composition alterations) [2]. This finding highlights the importance of integrating pre-harvest health management and timely postharvest measures for controlling chestnut rot [39].

Evaluation of natural decay incidence across the storage period and disease severity after artificial inoculation revealed significant resistance differences among the chestnut cultivars, indicating significant genetic differentiation existed in disease resistance among them [2]. Cultivar YJ consistently exhibited the highest disease severity in natural and artificial conditions, classifying it as a highly susceptible cultivar. Conversely, YG, YSSF, and DBH maintained the lowest decay incidence throughout storage, with YG showing the lowest disease index in the inoculation assay, indicating it possesses the strongest resistance. These resistance differences provide a direct theoretical basis for the selection and promotion of resistant cultivars. Furthermore, differences were observed in the degree of disease occurrence between natural and artificially inoculated nuts among several chestnut cultivars, suggesting there are distinct underlying resistance mechanisms or the effect of complex environmental conditions [41,42]. Natural decay incidence reflects the comprehensive resistance level of cultivar to multiple pathogens, whereas the artificial inoculation accurately characterizes the ability of resisting specific pathogens’ expansion [43]. YG showed the relatively lowest natural decay incidence and the lowest inoculation disease index, suggesting that it possesses advantageous physical structures and physiological or biochemical resistance mechanisms to possess both broad-spectrum and specific resistance [44,45].

Physical barriers, such as fruit firmness and waxy layer structure, limit pathogens’ infection, whereas nutrients like sucrose, sorbitol and fructose directly affect pathogens’ colonization and expansion [46,47]. In this study, correlation analysis revealed a significant negative correlation between the disease index and the contents of sucrose (SuC) and sorbitol (SoC), and a significant positive correlation with stachyose (StC) and fructose (FC). These associations may explain the different resistance across various chestnut cultivars following mixed inoculation. Future research could employ targeted metabolic engineering or exogenous application experiments to manipulate the levels of key sugars (e.g., SuC, SoC, StC, and FC) in specific cultivars, followed by fungal pathogens challenge, to directly test their functional roles in disease resistance. Mechanistically, these correlations may reflect a dual role of sugar metabolism in plant-pathogen interactions. Sucrose and sorbitol, as key osmoregulatory substances, energy sources and metabolic substrates, help maintain cellular vitality and enhance plant immunity when present at higher concentrations [48], thereby restricting pathogen spread. For instance, sucrose transporters SWEETs positively modulate sucrose synthesis and defense responses to enhance plant immunity [49]. Similarly, sorbitol accumulation has been linked to enhanced resistance, potentially via osmotic regulation and redox homeostasis, as the transcription factor MdWRKY79 regulates *MdNLR16* to promote sorbitol-modulated resistance against *A. alternata* [50]. Conversely, the positive correlation of fructose and stachyose with disease severity may indicate a metabolic reprogramming during infection [46]. The metabolic shift of sucrose and sorbitol toward fructose, stachyose and other soluble sugars by invertases—a process enhanced in pathogen-infected tissues [50,51]—may provide more suitable carbon sources for pathogen or mark a decline in fruit resistance [52]. Fruit firmness showed no significant correlation with the disease index, suggesting that physical barriers might not be a key resistance factor of chestnuts against these pathogens, which primarily invade through wounds. These findings highlight the potential of modulating kernel sugar composition— through breeding or postharvest interventions—as a promising strategy to enhance the disease resistance in chestnuts. Specifically, efforts could focus on (1) selecting or developing cultivars with inherently elevated sucrose and sorbitol levels, and (2) designing postharvest treatments that either maintain these beneficial sugar profiles or suppress the pathogen-triggered shift toward fructose accumulation. Future research directly testing the efficacy of such strategies through in vitro and controlled postharvest treatment trials will be essential to translate these mechanistic insights into practical control measures.

## 5. Conclusions

In this study, we identified six major pathogenic species—*D. eres*, *T. rugulosus*, *A. alternata*, *M. circinelloides*, *F. proliferatum*, and *R. stolonifer*—causing Chinese chestnut fruit rot in Hebei province in China. Further, we evaluated the disease resistance of six major cultivars and preliminarily revealed the relationship between soluble sugars and disease resistance in the nuts. These findings provide practical guidance for designing control strategies against chestnut fruit rot and for screening and breeding resistant cultivars. Future studies could focus on clarifying the infection mechanisms of these pathogens, uncovering physiological and biochemical resistance mechanisms across cultivars, and developing postharvest preservation technologies utilizing resistance-inducing substances.

## Data Availability

The original contributions presented in this study are included in the article/Supplementary Material. Further inquiries can be directed to the corresponding authors.

## Supplementary Materials

The following supporting information can be downloaded at https://www.mdpi.com/article/10.3390/microorganisms14010113/s1. Table S1: Gene regions and primers used in this study; Table S2: Isolates and GenBank accession numbers used for *Diaporthe* genera in this study; Table S3: Isolates and GenBank accession numbers used for *Talaromyces* genera in this study; Table S4: Isolates and GenBank accession numbers used for *Alternaria* genera in this study; Table S5: Isolates and GenBank accession numbers used for *Mucor* and *Rhizopus* genera in this study; Table S6: Isolates and GenBank accession numbers used for *Fusarium* genera in this study; Table S7: The data on decay incidence in six chestnut cultivars during cold storage; Table S8: The data of disease index, soluble sugar content and firmness used for correlation analysis.
