# Supplementary material for: Identification of Fungal Pathogens of Chinese Chestnut Fruit Rot and Analysis of Resistance Differences Among Major Cultivars
Source: Microorganisms. 2026 Jan 5;14(1):113. doi: 10.3390/microorganisms14010113 (PMC12844437; doi:10.3390/microorganisms14010113)
Supplement: Supplementary file 1 [file microorganisms-14-00113-s001.zip › microorganisms-4046142-supplementary.pdf]

Table S1. Gene regions and primers used in this study.

Gene Region	Primer Pairs	Sequences (5'-3')	Annealing temperature (°C)	Reference
ITS	ITS4/ITS5	TCCTCCGCTTATTGATATGC/ GGAAGTAAAAGTCGTAACAAGG	55	[17]
TUB2	Bt2a/Bt2b	GGTAACCAAATCGGTGCTGCTTTC/ ACCCTCAGTGTAGTGACCCTTGGC	60	[18]
TEF1-α	EF1-728F/EF1-986R	CATCGAGAAGTTCGAGAAGG/ TACTTGAAGGAACCCTTACC	54	[17]
CAL	CAL-228F/ CAL-737R	GAGTTCAAGGAGGCCTTCTCCC/ TGRTCNGCCTCDCGGATCATCTC	54	[19]
HIS	CYLH3F/ H3-1b	ACTAAGCAGACCGCCCGCAGG/ GCGGGCGAGCTGGATGTCCTT	57	[18]
RPB2	RPB2-5F2/ RPB2-7cR	GGGGWGAYCAGAAGAAGGC/ CCCATRGCTTGTYYRCCCAT	55	[20]
GAPDH	GPD1/GPD2	CAACGGCTTCGGTCGCATTG/ GCCAAGCAGTTGGTTGTGC	55	[20]

Note: ITS, internal transcribed spacer regions 1 and 2 and intervening 5.8S nrDNA; *TUB2*, beta-tubulin gene region; *TEF1-α*, translation elongation factor 1-alpha; *CAL*, calmodulin; HIS, histone H3; *RPB2*, RNA polymerase second largest subunit; *GAPDH*, glyceraldehyde 3-phosphate dehydrogenase.

Table S2. Isolates and GenBank accession numbers used for *Diaporthe* genera in this study.

Species	Strain/Isolate	ITS	*TUB*	*TEF*	*CAL*	*HIS*
Diaporthe alnea	CBS 146.46	KC343008	KC343976	KC343734	KC343250	KC343492
D. anacardii	CBS 720.97	KC343024	KC343992	KC343750	KC343266	KC343508
D. batatas	CBS 122.21	KC343040	KC344008	KC343766	KC343282	KC343524
D. camelliae-sinensis	SAUCC194.92	MT822620	MT855817	MT855932	MT855699	MT855588
D. chamaeropis	CBS 753.70	KC343049	KC344017	KC343775	KC343291	KC343533
D. eres	**BL-1**	**PX597157**	**PX526137**	**PX526138**	**PX526139**	**PX526140**
	AR5193	KJ210529	KJ420799	KJ210550	KJ434999	KJ420850
	DP0667	KJ210524	KJ420790	KJ210548	KJ435009	KJ420840
	FAU506	KJ210526	KJ420792	JQ807403	KJ435012	KJ420842
D. grandiflori	SAUCC194.84	MT822612	MT855809	MT855924	MT855691	MT855580
D. heterophyllae	CPC 26215	MG600222	MG600226	MG600224	MG600218	MG600220
D. heliconiae	SAUCC194.77	MT822605	MT855802	MT855917	MT855684	MT855573
D. litchii	SAUCC194.22	MT822550	MT855747	MT855863	MT855635	MT855519
D. neilliae	CBS 144.27	KC343144	KC344112	KC343870	KC343386	KC343628
D. nomurai	CBS 157.29	KC343154	KC344122	KC343880	KC343396	KC343638
D. shennongjiaensis	CNUCC201905	MN216229	MN227012	MN224672	MN224551	MN224560
D. subclavata	SAUCC194.66	MT822594	MT855791	MT855906	MT855674	MT855562
Diaporthe alnea	CBS 146.46	KC343008	KC343976	KC343734	KC343250	KC343492
D. anacardii	CBS 720.97	KC343024	KC343992	KC343750	KC343266	KC343508
D. batatas	CBS 122.21	KC343040	KC344008	KC343766	KC343282	KC343524
D. camelliae-sinensis	SAUCC194.92	MT822620	MT855817	MT855932	MT855699	MT855588

Note: Isolates marked in bold and blue are new sequences in this study.

Table S3. Isolates and GenBank accession numbers used for *Talaromyces* genera in this study.

Species	Strain/Isolate	ITS	*TUB*	*RPB2*
Talaromyces rugulosus	**BL-2**	**PX597158**	**PX526141**	**PX526142**
	CBS371.48	KF984834	KF984575	KF984925
T. atricola	CBS255.31	KF984859	KF984566	KF984948

T. scorteus	NRRL 62676	KX657328	KX657079	KF984916
T. tratensis	CBS113146	KF984891	KF984559	KF984911
T. rotundus	CBS:369.48	MH856398	JX494291	KM023275
T. tardifaciens	CBS 250.94	JN899361	KF984560	KF984908
T. allahabadensis	CBS:453.93	MH862430	KF984614	KF985006
T. radicus	CBS:100489	MH862702	KF984599	KF985013
T. loliensis	CBS:643.80	MH861302	KF984658	KF985021
T. islandicus	CBS 338.48	JN899318	KF984655	KF985018
T. brunneus	CBS:227.60	MH857960	JX494296	KM023272
T. emodensis	CBS:100536	MH862707	KJ865724	JF417445
T. proteolyticus	CBS 303.67	JN899387	KJ865729	KM023301
T. mimosinus	CBS:659.80	MH861304	KJ865726	MN969149
T. bacillisporus	CBS:296.48	MH856351	AY753368	JF417425
T. palmae	CBS 442.88	JN899396	HQ156947	KM023300
T. subinflatus	CBS 652.95	JN899397	MK450890	KM023308

Note: Isolates marked in bold and blue are new sequences in this study.

Table S4. Isolates and GenBank accession numbers used for *Alternaria* genera in this study.

Species	Strain/Isolate	ITS	*GAPDH*	*TEF1*	*RPB2*
Alternaria alternata	**BL-3**	**PX597159**	**PX526143**	**PX526144**	**PX526145**
	CBS102598	KP124329	KP124184	KP125105	KP124797
	CBS118814	KP124357	KP124211	KP125133	KP124825
A. arborescens	CBS102605	AF347033	AY278810	KC584636	KC584377
A. avenicola	CBS 121459	KC584183	KC584100	KC584639	KC584380
A. burnsii	CBS 107.38	KP124420	JQ646305	KP125198	KP124889
A. brassicicola	CBS 118699	JX499031	KC584103	KC584642	KC584383
A. eichhorniae	CBS 489.92	KC146356	KP124276	KP125204	KP124895
A. gaisen	CBS 118488	KP124427	KP124278	KP125206	KP124897
A. gossypina	CBS 104.32	KP124430	JQ646312	KP125209	KP124900
A. iridiaustralis	CBS 118486	KP124435	KP124284	KP125214	KP124905
A. jacinthicola	CBS 133751	KP124438	KP124287	KP125217	KP124908
A. longipes	CBS 540.94	AY278835	AY278811	KC584667	KC584409
A. infectoria	EGS 27-193	DQ323697	AY278793	KC584662	KC584404
A. papavericola	CBS 116606	FJ357310	FJ357298	KC584705	KC584446
A. solani	CBS 109157	KJ718238	GQ180080	KJ718585	KJ718413

Note: Isolates marked in bold and blue are new sequences in this study.

Table S5. Isolates and GenBank accession numbers used for *Mucor* and *Rhizopus* genera in this study.

Species	Strain/Isolate	ITS
Mucor amethystinus	CBS526.68	JN206015
	CBS202.28	NR_169895
M. bainieri	CBS293.63	NR_103628
M. brunneogriseus	CBS129.41	MH856086
M. circinelloides	**BL-4**	**PX597160**
	CBS195.68	HQ154604
M. ctenidius	CBS293.66	MH858796
M. griseocyanus	CBS116.08	NR_126136
M. janssenii	CBS205.68	NR_126123
M. lusitanicus	CBS108.17	NR_126127
M. phayaoensis	CN040I1	PP956629
M. pseudolusitanicus	CBS543.80	MF495060
M. racemosus	SB7	MN944889

M. ramosissimus	CBS135.65	NR_103627
M. variicolumellatus	CBS236.35	JN205979
M. velutinosus	ATCC MYA-4766	NR_111682
Rhizopus americanus	CBS340.62	HM999967
R. arrhizus	CBS130145	MH865594
R. caespitosus	CBS427.87	AB097387
R. delemar	MU1	KY628942
R. homothallicus	CBS336.62	AB097388
R. koreanus	CNUFC_EML-HO95-1	NR164543
R. lyococcus	CBS319.35	AB100449
	CBS117.43	JN206375
R. microsporus var. chinensis	CBS631.82	AB097394
R. microsporus var. rhizopodiformis	CBS536.80	AB097390
R. microsporus var. oligosporus	CBS337.62	AB097395
R. microsporus var. microsporus	CBS699.68	AB097385
R. oryzae	CBS112.07	AB097334
R. schipperae	CBS138.95	HM999969
R. schipperae	ATCC96514	AB106340
R. sexualis	CBS336.39	AB113016
R. stolonifer	**BL-6**	**PX597162**
	CBS150.83	AB113022
	CBS609.82	AB113023

Note: Isolates marked in bold and blue are new sequences in this study.

Table S6. Isolates and GenBank accession numbers used for *Fusarium* genera in this study.

Species	Strain/Isolate	ITS	*RPB2*	*TEF1*
Fusarium contaminatum	CBS111552	-	MH484900	MH484991
F. glycines	CBS176.33	-	MH484868	MH484959
F. globosum	CBS428.97	-	MT010982	MT010993
F. gossypinum	CBS116611	-	MH484907	MH484998
F. languescens	CBS645.78	-	MH484880	MH484971
F. langsethiae	NRRL54940	-	MW233482	MW233138
F. libertatis	CPC25782	-	MH484932	MH485023
F. mangiferae	NRRL25226	-	JX171622	AF160281
F. nirenbergiae	CBS129.24	-	MH484864	MH484955
F. oxysporum	CBS144134	-	MH484953	MH485044
F. proliferatum	**BL-5**	**PX597161**	**PX526146**	**PX526147**
	GR_FP172	-	MT305150	MT305208
F. sambucinum	FRC_R4712	-	MW233492	MW233148
F. spartum	NRRL66894	-	MT409447	MT409457
F. triseptatum	CBS116619	-	MH484910	MH485001
F. udum	CBS177.31	-	MH484866	MH484957
F. venenatum	FRC_R-09186	-	GQ915499	GQ915515

Note: Isolates marked in bold and blue are new sequences in this study. “-” means no sequence information of the corresponding strain/isolate.

Table S7. The data of decay incidence in six chestnut cultivars during cold storage.

	Decay rate /%						
	0 d	30 d	60 d	90 d	120 d	150 d	180 d
Yanshan Shuofeng (YSSF)	0.33±0.24 c	0.22±0.31 c	0.50±0.41 c	1.24±0.72 c	1.94±0.73 c	3.73±1.11 d	3.88±1.20 c
Yanshan Zaofeng (YSZF)	1.83±0.24 b	2.28±0.97 b	4.28±2.17 b	8.06±1.86 b	10.75±3.49 b	20.31±2.82 b	25.67±2.62 b
Yanjing (YJ)	3.36±0.66 a	5.01±0.50 a	8.67±1.49 a	13.33±1.44 a	19.99±1.10 a	30.83±1.76 a	47.71±4.94 a
Yanguang (YG)	0.33±0.27 c	0.27±0.22 c	0.36±0.27 c	0.73±0.55 c	1.15±0.29 c	1.38±0.17 d	1.67±0.57 c
Yanli1 (X19-94)	1.33±0.24 b	2.60±0.47 b	4.02±1.21 b	5.97±0.41 b	10.66±2.58 b	12.91±1.90 c	20.64±1.53 b
Dabanhong (DBH)	0.44±0.42 c	0.46±0.33 c	0.64±0.39 c	1.94±0.34 c	2.98±0.41 c	3.93±0.32 d	5.27±0.66 c

Note: Data are expressed as the mean ± SD. Different letters in each storage period indicate significant differences at $p < 0.05$.

Table S8. The data of disease index, soluble sugars content and firmness used for correlation analysis

	Disease index (DI)	Soluble solid content (SSC) /%	Water content (WC) /%	Amylose content (AC) /%	Starch content (SC) /%	Total soluble sugar content (TSS) /%	Sucrose content (SuC) /%	Maltose content (MalC) /%	Stachyose content (StC) /%	Fructose content (FC) /%	Mannitol content (ManC) /%	Sorbitol content (SoC) /%	Fruit firmness (FF) /%	Kernel firmness (KF) /%
Yanshan	49.74	23.51	0.49	0.38	0.62	0.18	7.59	0.37	0.18	0.92	0.05	0.05	65.99	23.93
Shuofeng	45.56	24.12	0.49	0.37	0.63	0.21	7.86	0.39	0.18	0.85	0.05	0.05	48.24	24.13
(YSSF)	51.31	25.17	0.50	0.38	0.62	0.23	7.95	0.40	0.19	0.77	0.05	0.05	72.79	18.44
mean±SD	48.87±2.97	24.27±0.83	0.49±0.01	0.38±0.01	0.62±0.01	0.21±0.03	7.80±0.19	0.38±0.02	0.18±0.01	0.85±0.01	0.05±0.00	0.05±0.01	62.34±12.68	22.17±3.23
Yanshan	52.38	22.89	0.47	0.42	0.58	0.14	9.3	0.40	0.21	0.93	0.03	0.05	51.85	21.36
Zaofeng	48.72	24.01	0.51	0.37	0.63	0.19	8.58	0.46	0.17	0.89	0.04	0.04	44.79	20.82
(YSZF)	51.39	24.01	0.51	0.4	0.6	0.18	8.34	0.51	0.18	0.83	0.05	0.03	60.11	16.92
mean±SD	50.83±1.89	23.64±0.65	0.50±0.02	0.40±0.03	0.60±0.03	0.17±0.03	8.74±0.50	0.46±0.06	0.18±0.02	0.88±0.05	0.04±0.01	0.04±0.01	52.25±7.67	19.70±2.42
Yanjing	63.35	23.42	0.49	0.36	0.64	0.17	7.02	0.39	0.21	1.03	0.04	0.04	92.27	24.91
(YJ)	77.78	21.83	0.49	0.35	0.65	0.17	6.78	0.40	0.20	1.03	0.05	0.03	85.04	23.07
	70.37	21.45	0.49	0.34	0.66	0.16	6.84	0.39	0.20	1.03	0.05	0.03	87.05	28.52
mean±SD	70.50±7.22	22.23±1.05	0.49±0.00	0.35±0.01	0.65±0.01	0.17±0.01	6.88±0.12	0.39±0.01	0.20±0.01	1.03±0.01	0.05±0.01	0.03±0.01	88.12±3.73	25.50±2.77
Yanguang	33.33	25.31	0.48	0.38	0.62	0.17	9.78	0.39	0.10	0.75	0.04	0.04	66.61	24.61
(YG)	33.33	26.85	0.47	0.38	0.62	0.20	10.08	0.39	0.09	0.76	0.05	0.05	72.00	22.17
	32.22	24.44	0.50	0.39	0.61	0.18	9.84	0.40	0.10	0.75	0.04	0.05	69.34	26.12
mean±SD	32.96±0.64	25.53±1.22	0.48±0.03	0.38±0.01	0.62±0.01	0.18±0.02	9.90±0.16	0.39±0.01	0.10±0.01	0.75±0.01	0.04±0.01	0.05±0.01	69.32±2.70	24.30±1.99
Yanli1	58.97	20.42	0.49	0.46	0.54	0.14	6.84	0.51	0.17	0.73	0.04	0.03	68.33	22.01
(X19-94)	57.33	24.86	0.50	0.37	0.63	0.15	7.02	0.51	0.16	0.73	0.04	0.03	60.88	23.73
	55.56	21.92	0.49	0.37	0.63	0.21	6.96	0.52	0.16	0.73	0.05	0.03	45.75	23.66

mean±SD	57.29±1.71	22.40±2.26	0.49±0.01	0.40±0.05	0.60±0.05	0.17±0.04	6.94±0.09	0.51±0.01	0.16±0.01	0.73±0.00	0.04±0.01	0.03±0.00	58.32±11.51	23.13±0.97
Dabanhong	54.74	28.01	0.44	0.41	0.59	0.21	9.12	0.44	0.23	0.90	0.05	0.04	79.12	22.33
(DBH)	55.07	28.44	0.47	0.49	0.51	0.20	9.24	0.44	0.23	0.89	0.05	0.05	80.70	23.48
	48.02	27.35	0.47	0.43	0.57	0.22	9.12	0.44	0.23	0.89	0.05	0.04	67.44	20.89
mean±SD	52.61±0.55	27.93±0.55	0.46±0.02	0.44±0.04	0.56±0.04	0.21±0.01	9.16±0.07	0.44±0.00	0.23±0.00	0.89±0.01	0.05±0.00	0.04±0.01	75.75±7.24	22.23±1.30

Note: These data were measured before in our previous study [24].
